# Exploring the Motivational Factors for International Students to Study in Chinese Higher Education Institutions

**DOI:** 10.3389/fpsyg.2022.938986

**Published:** 2022-07-07

**Authors:** Fakhra Yasmin, Shengbing Li, Gabriela Slaninová

**Affiliations:** ^1^School of Education, South China Normal University, Guangzhou, China; ^2^Department of Management, Faculty of Informatics and Management, University of Hradec Kralove, Hradec Kralove, Czechia

**Keywords:** international students, motivation, higher education, Chinese universities, ANOVA

## Abstract

China has witnessed a remarkable surge in the enrollment of international students in recent years and the state government has made a massive investment to build key universities of international repute. These trends made it imperative to investigate the underlying motivational aspirations of foreign students arriving from diverse regions to get enrolled in public sector Chinese universities. The present study designed an in-depth survey questionnaire and collected data from 618 foreign students enrolled in postgraduate programs at seven key state universities in the Hubei province of China. The item-based, dimension-based, and variable-level analysis approach is used to systematically uncover the facets of the internationalization of Chinese higher education in the current setting. In so doing, we employ descriptive statistics, principal component analysis, ANOVA, correlation and regression estimations, and path models to ensure the robustness of empirical outcomes. In light of the push and pull factor model regarding motivational factors for foreign students to study in China, the findings of this study assert that academic pursuits mainly dictate the international student’s decision to attain higher education in China. While obtaining a foreign degree, international image prestige, and better employment prospects after the completion of the degree were the key intentions that mainly shape the students’ decision to get enrolled in Chinese universities. Furthermore, the discriminant analysis posits that international students significantly differ in their motivational dimensions to obtain a higher degree from China. As foreign students from Asia and Africa mainly have academic goals while Europeans and Americans predominantly have pleasure and enjoyment motives to study in China. The outcomes of this research can assist Chinese administrators to understand the key motivational factors for foreign students to study in China and devise a policy accordingly to attract high quality foreign talent.

## Introduction

Since the adoption of the reformation and official launch policy in 1980, the phenomenal rise of China’s economy has piqued the interest of many countries when it comes, to commercial, and educational cooperation. International students’ mobility is a cornerstone of internationalization around the globe. Accordance to the Organization of Economic Corporation and Development (OECD), “the global demand for international higher education is set to grow by 7.2 million students by 2025.” Countries and institutions must satisfy these students’ aspirations in order to compete in the global market by creating rigorous strategies and policies that include a financial investment in the students’ enrollment (James and Yun, 2017). The active mobilization of students embarked on demographic, economic, and socio-linguistic structures (Li et al., 2020). Since 2011 China became the world’s second-largest economy, Because of its economic strength and expanding home market, it has played a unique position as a hub for regional commerce, economic growth, and integration into Asia and Africa (Xing, 2019).

Globalization of education in China went through several stages in accordance with globalization trends and to strengthen cultural, political, and economic relations between China and the rest of the world. By 2018, about 500,000 international students were obtaining higher education from China, with 70% of them coming from Africa and Asia (Ministry of Education of the People’s Republic of China, 2020). According to the most recent scenario, the Chinese government’s international education plan for the decade 2010–2020 aimed to gradually increase the number of foreign students, and by 2020, the government plans to accept more than 500,000 foreign students, 150,000 who are now expected to be research students enrolled in graduate and postgraduate programs. In order to meet these targets, Chinese institutions accelerated the enrollment of scholarship and self-finance students, and China enrolled 397,635 overseas students in 2015, making it Asia’s largest host country (MOE, 2016; Xing, 2019).

The attainment of education specifically higher education in a foreign country is becoming an educational trend in recent times which is perhaps the reason that the worldwide mobility of students has increased dramatically (Vázquez et al., 2014). The students who decide to pursue foreign education have to experience difficulties in adjustment and adaptation to a new environment. Mainly adjustment and adaptation problems were related to psychological, educational, and socio-cultural issues. Despite these concerns, the inclination toward foreign education is rising exponentially mainly because of accessible resources, equitable opportunities, and flexibility in educational policies. It motivates the students to be enrolled in foreign educational programs that lead to short-term stay abroad or permanent migration. The reasons or motivational factors behind foreign mobility are not explored much, particularly about deciding to go abroad, stay and study there (Lauermann, 2015).

Students’ choice to study abroad is considered as a first stride toward the most important revolution in their academic career mainly for those whose degree necessitates staying for an extended time period (Ward et al., 2020). The decision to study abroad implies an educational experience in a foreign academic system that generally leads to an advancement in the academic stage like from graduate to postgraduate stage. On top of it, this decision entails facing challenges of a different and new environment, sociocultural change, and psychological efforts of adaptation (Selvarajah, 2006). The term “motivation” in this perspective is explained as every effort or activity undertaken for the decision to acquire foreign education may become a reason for students’ choice to foreign study. The factors of motivation mainly consisted of values, goals, and expectations. Hence, students generally to decide study abroad for the fulfillment of their goals or to accomplish their values and expectations. Educational policies and students’ academic backgrounds also work as motivational factor that affects the students’ study abroad decisions (Lauermann, 2015). Likewise Gbollie and Gong (2019) observed that availability of scholarship, easy visa entry requirements, and perception of quality education are the important push and pull factors for foreign students to opt China as their study destination.

Students have a variety of reasons for studying abroad like job opportunities, culture, social understanding, and immigration. Min et al. (2012a) classified students’ motivation for study abroad into two main categories. The first one is acquiring quality education and the second is looking for better career opportunities such as jobs, immigration chances, settlement, and to broaden exposure and social experience. Chirkov et al. (2007) recognized two main objectives of students that motivate them to study abroad that are preservation objective and self-development objective. The preservation objective comprises activities and efforts that were undertaken to escape from problems in the home country to ensure one’s safety. While self-development objective includes endeavors such as getting a quality education and better job opportunities. Chirkov et al. (2008) identified that students who independently decided to study abroad for higher education and settlement purposes were much more satisfied and successful as compared to those who moved abroad unwillingly and merely due to the pressure of family, and people, or society.

China is the most attractive platform for international students. China’s international higher education department is a global player worthy of attention and is changing rapidly in a fast-growing economy (Peters et al., 2020). The Chinese government is spending millions of dollars in terms of grants and scholarships to foreign students as the government provided scholarships to 40,600 foreign students enrolled in degree programs in 2015 which makes up for 89.38% of total degree students studying in China (MOE, 2016). This shows massive investment on the part of the Chinese government in an attempt to internationalize its educational environment. Among the very aims of the internationalization plan implemented by the government, creating some top-ranked universities in various disciplines by inviting the world talent, upsurge innovation, and research productivity in order to transform China from a knowledge user to a knowledge-producing country and bringing Chinese higher education system at par with the developed world are some of the important objectives that the government aspire to achieve through these educational investments. Furthermore, the Ministry of Education of the People’s Republic of China (2020) reports that in 2018, China had over 492,000 students from 192 countries studying in 31 provinces, with not all of them receiving government or institutional funding for their studies. This depicts that China is now becoming the most powerful market for international students. Besides, the inbound foreign students in China were found to bring in economic benefits by significantly boosting foreign direct investment (Lin et al., 2020).

In the backdrop of these assertions, it is important to examine the motives of foreign students coming to China specially to pursue a graduate or postgraduate degree program. The contribution of this research lies in the fact that it empirically examines both academic and non-academic motives of international students to opt China as their study destination. The outcomes of such investigation will assist Chinese HEIs in identifying the underlying aims of foreign students hence enabling them to enhance their particular services to ensure a more fulfilling learning experience. In addition, as postulated by previous researchers that pre-enrollment motivation can influence the level of satisfaction of the students (Yasmin et al., 2021), the present study investigates whether foreign students with a higher level of motivation to pursue their studies in Chinese HEIs experience higher satisfaction and vice versa. The current drift of literature uses survey research methods to explore the motivational factors, especially for Master’s and Ph.D. degree students to study in the Chinese host universities. For determining the key motives behind foreign students’ motivation to select China as their study destination and their perceived degree of satisfaction, and adapted Five points Likert scale was used to obtain the responses of foreign students. A total of 618 questionnaires were collected and analyzed from Masters and doctorate students enrolled in the seven public sector universities of Wuhan, China. Respondents respond to the dimensions of academic and education quality, personal and financial security, career and migration, pleasure and experience, and other motivational factors.

The contribution of this manuscript is twofold. China has emerged as an international education market. It has overtaken the United Kingdom and it is the second-largest host of international students’ population (Cai, 2020). Firstly, a large stream of literature in the context of China focused on the satisfaction level of foreign students with the studies in China but has ignored the evaluation of the motivational factors for the foreign students to study in Chinese Universities. In view of the increased incursion of China in regional and world political and economic scenarios and a massive influx of international students in China in recent years makes it is imperative to know about the motivation of students in choosing China as a study destination and their current experience and satisfaction while studying in China. Notwithstanding, China is a vast international education market and there is a rapid influx of international students, it is imperative to investigate the motivational factors to figure out the sustainability of the Chinese international higher education system. Hence, this study is an endeavor to construct a bridge between the stream of foreign students in China and the sustainability of the Chinese international higher education system by determining the factors that motivate these students to study in Chinese universities. Secondly, to ensure the accuracy of this study, we use a bottom-up approach by using descriptive, reliability, principal component analysis (PCA), and analysis of variance (ANOVA) techniques to conduct the analysis.

The rest of the paper is constructed as follows. Section “Literature Review” discussed the theoretical underpinnings and literature review. The research methodology is discussed in section “Materials and Methods”. Section “Data Analysis and Interpretation” depicts the results and discussion. Conclusion along with policy implications and suggestions are given in section “Conclusion”.

## Theoretical Framework and Literature Review

### Literature Review

Several motivational theories such as the expectations framework, cognitive and social theory, and need-based theories like self-determination or autonomy theory resolve the ambiguities in acquiring foreign education. Despite concentrating on particular goals or reasons to acquire foreign education, these theories reflect the more general principles that lead the students toward varied academic opportunities or selections (Wigfield and Eccles, 2000). As per the expectations framework, these principles just consist of the interaction among expectations of students to succeed if the students avail the given opportunity such as acquiring foreign education and values of students linked with this selection like cost-benefit analysis that involve efforts to increase the job chances because of foreign education and accomplishment value such as self-efficacy (Eccles and Wigfield, 2003). This framework supplements the prior-reviewed research in several ways. Firstly, the values and expectations theory is very crucial to determining the decision-making of students. This suggests that although the majority of the students significantly value the study abroad as the result of the push/pull model, the students will not prefer to be engaged in foreign education programs if they have any doubt of failure. It provides further insight about push/pull factors that these factors may not be sufficient to describe the individual decision-making of students’ consideration to acquire a foreign degree (Mazzarol and Soutar, 2002; Macready et al., 2011).

Secondly, however, the expectation factor is usually neglected; several push/pull factors and identification of goals related to studying abroad are relevant to the value aspects of the discussed framework. This involves such as perception of cost (expenses or funds), usability (acquisition of quality education and job chances), and concentration or interest (the availability of chance to know and experience the diverse culture) (Mazzarol, 1998; Wilkins et al., 2011). Thirdly, the pre-enrollment expectations theory is also related to this viewpoint. For example, Goldstein and Kim (2006) claimed that the components of the value system like expected pleasure and joy from the foreign education experience and the expectancy to interact with diverse people are the positive predictors of the foreign education program. The expectation of success is a very important factor in the theory of value expectancy as well as in the theory of social cognition. According to social cognition theory, academic choices draw a solid impact and have a strong influence on the supposed self-efficacy of students.

The self-efficacy of students is the perceived competence to perform a required course of action to accomplish a particular motive or goal. Thus, people with a higher level of self-efficacy have a strong belief in their abilities to succeed. These people are likely to establish quite tough and stimulating tasks and are much interested in spending more effort and energy to overcome the difficulties. This attitude increases performance and success (Bandura, 1997). At the same time, low self-esteem leads to avoiding behavior such as the decision to stay in the home country despite having an interest to go abroad for studies. It is merely due to less trust in one ability that results in fear of failure inside. The significant antecedents of self-efficacy contain previous experience of achievement and failure, the effect of relevant societal orientation (such as perception of other people’s successful experience of studying abroad) social inducement like the positive and encouraging attitude of parents, and psycho-physical responses like nervousness and fears associated with stay and study in a foreign country (Lee, 2008).

Similar to the expected value framework, the low level of self-efficacy related to foreign education describes the reasons for deciding not to move abroad for studies even with the presence and effect of push/pull factors. The students’ age was also found influential in the decision making to travel abroad for study purposes (Simpson, 2010). Furthermore, this framework supplements the previous literature because numerous factors that describe the “inclination to foreign education” also link with antecedents of self-efficacy. It also involves previous education success and linguistic abilities, socio-cultural paradigm, the influence of parents and background of the family, and emotional responses to the varied cultural interactions (Goldstein and Kim, 2006; Salisbury et al., 2008). Thus, considering the influence of these antecedents, the concept of acquiring foreign education explained by the self-efficacy theory may be perceived as the mechanism of motivation that links with the process of personal decision to move abroad for studies. These links among antecedents and significance of motivational concepts establish the basic merits of research that deal with theories of motivation, as opposed to the models that focused only single concept. As well as it permits the researcher to establish theory-based assumptions to examine a blend of theoretically associated factors such as value-expectancy. Moreover, these theoretical paradigms can also help to establish significant mediation by creating scenarios that encourage the basic requirements of independence, competency, and association of individuals.

The international students’ decision to study in a foreign country has been examined from several viewpoints. In the context of the educational market, the leading framework to identify the factors is known as “push and pull” factors. It is believed that the push and pull factors are probably more important in attracting international students to a specific institution or country (McMahon, 1992; Mazzarol and Soutar, 2002). Some supplementary approaches incorporate some other factors like the university selection model (Lee, 2008; Salisbury et al., 2008), studies on students’ beliefs, pre-enrollment expectations before selecting any country (Chirkov et al., 2007), and their goals and motivations (Martin et al., 1995; Chirkov et al., 2008) before deciding to move abroad. These frameworks provide important perspectives of motivation to understand the reason for students’ decision of taking foreign education. They basically draw attention to some particular motivational aspects rather than based on theoretical grounds of psychology. Amongst the aforementioned models, the push and pull factors model more accurately elucidate the motivational elements for foreign students to study in Chinese HEIs and hence provides the conceptual basis for the empirical inquiry of this study.

The most prevalent and frequently used model of the foreign relocation of students to study abroad is based on the description and implications of “push and pull” factors (Altbach, 1998). Maringe and Carter (2007) described that mainly the process of students’ decision making regarding studying abroad is influenced by the blend of push and pull factors. Push factors are based on the situations from which the individuals try to escape in their own country like inadequate academic facilities, economic, social, and security issues, etc. While pull factors are grounded on the individuals’ preferred situations related to the foreign or host countries like a high level of academic quality and a better standard of living (Macready et al., 2011). The push factors strongly influence the overall decision of students to get enrolled in an abroad study program. Whereas, given the available opportunities, pull factors play a vital role in the choice of a specific country as a study destination (Mazzarol, 1998).

Another version of the push and pull model is a three-stage model which was introduced by Mazzarol and Soutar (2002). The model describes the decision-making phases of students to get registered in an overseas study program. The primary stage of the model represents that students’ decision to foreign education rather than studying in their home country is shaped by the push factors. The second stage designates that students’ selection of a particular country as their study destination is determined by pull factors. Henceforth, taking into consideration the available options, the selection of a specific institution for higher education from the host country is the last step in the decision-making process to study abroad. The push and pull factors are diverse. For instance, push factors are typically associated with inadequate academic opportunities and improper educational access in the home country (Li and Bray, 2007), and social and financial dissatisfaction in the domestic country (Mazzarol and Soutar, 2002; Macready et al., 2011). While pull factors apply to the recommendation and support provided by friends and family to move to study abroad, the host country’s repute, economic matters like fees and living expenses during the stay abroad, and social concerns like discriminatory attitudes and style of living (Mazzarol and Soutar, 2002; Li and Bray, 2007).

The review of past studies in this area proposes that the three-stages push and pull factor model has been adopted in numerous researches, especially for students from Asian and African countries. The most significant push factors identified by the empirical studies are academic and job dissatisfaction arising from inadequate educational opportunities at home and inclination of the job market toward individuals holding a foreign degree (Wilkins et al., 2011). Additionally, the influence and pressure of family is another noteworthy factor in deciding to study abroad. The economic and political unrest in the domestic country is also a common issue (Pimpa, 2003). While, the most common pull factors are relevant to the provision of quality education, abundant employment opportunities, and a chance to be acquainted with a different culture in the host country. Besides, some scholars attempted to transform various recognized factors into organized categories, for instance, the category of social and cultural factors encompasses similarities between the home and host country and geographical and cultural intimacy. The category of economic factors includes matters relevant to educational fees and living expenses in the host country. The category of political factors involved educational policy establishment and academic support (Naidoo, 2016). Though the vast majority of research is concentrated on a particular factor as compared to diverse categories and different subsections of that factor have been explored to understand the student’s decision process.

### Literature Review

There has been a significant emphasis given to providing the students with a broader interaction with the global business community over the past few years. The academic institutes acknowledged this situation by offering students different opportunities to seek foreign education especially by increasing the availability of various short-term and long-term programs to study overseas (Mills et al., 2010). There are multiple prospects available for the students to move abroad for pursuing education. Specifically, the increasing number of opportunities for international study programs provides social and economic benefits to the less privileged students. Students’ participation in overseas study programs was found associated with an increase in their confidence and global understanding (Slotkin et al., 2012).

The study abroad programs may be short-term or long-term in nature. The short-term study abroad programs mostly involve students’ movement for one or more than one semesters to a host country or to spend a summer in the host country without being entitled to complete the entire degree. The popularity of short-term study programs abroad has markedly increased and the reasons behind such a surge are perhaps the resources and economic issues confronted by the universities and colleges and also the budgetary limitations faced by the students as well as their families to afford a full-time long-term study program (Carley et al., 2011). The students in long-term study abroad programs are likely to be enrolled in master’s or post-graduate degree programs to advance their qualifications in the host country. In recent times, there is another popular form of international education, such as studying on an international campus which is sited in the home country. This form of education provides foreign degrees to their students without students being spending their whole time abroad but are required to spend a limited time on the main campus located in the foreign country (Wilkins et al., 2011).

There is another important difference among students who take part in foreign study programs. This difference can be termed as some students have got any form of scholarship programs while others are known as the free movers and they are independent in organizing their study based on their own resources. An important and comprehensive study was conducted in Germany based on international students’ motives to be there. The study findings posit that the majority of international students were free movers. These students mainly came from developing countries with a motive to migrate to a developed country and be enrolled in long-term study programs like post-secondary or postgraduate programs to expand their stay abroad. The free movers have three interesting distinctions. Firstly, these students have no financial support and guidance from the study abroad program. What is more, due to their long-term stay abroad they have to face challenging situations in their academic, psychological and sociocultural pursuits, therefore, completion of a degree becomes difficult for such students. Lastly, these students are the main target of the educational market because they are self-funded and open to relocation to any other country (Bhandari and Blumenthal, 2011).

The inclination amongst various forms of mobility is normally linked with international students’ regions from which they belong. This background of international students affects their goals and motivations for the foreign study program. Lauermann (2015) cited that foreign students who come from developed and advanced countries are mostly involved in the short-term academic program while foreign students who engage in long-term educational programs are usually from developing or less economically advanced countries. In addition, studies found that students from advanced countries do not only prefer to undertake short-term educational programs over long-term educational programs but they also prefer social goals over academic goals (Mazzarol and Soutar, 2002).

Likewise, Llewellyn-Smith and McCabe (2008) evaluated the study abroad motives of international students registered in an international student exchange program in Australia by taking a sample from America and Europe. The results showed that availing the opportunity for enjoyment, fun, travel, desire to experience the changing weather, natural climate, and tourism are the reasons for mobility. Moreover, contrasting to the previous research on international students from less advanced and developing countries, the motive for career enhancement by studying abroad was not highly considered to be an important goal by these student’s goals (Mazzarol and Soutar, 2002). The students taking part in short-term or exchange programs are considered educational tourists (Llewellyn-Smith and McCabe, 2008).

Another study comprised of international students from North and Latin America and Europe was conducted in Mexico. The outcomes of this study pointed out that the students from Europe and North America had shown their interest in the short-term study program and were generally excited about gaining study experience in Mexico rather than getting a degree. While the Latin American students had possessed high educational goals and were committed to completing the degree and also wanted to upgrade their academics (Cantwell et al., 2008).

In general, the above-discussed studies entail that the international students’ region plays a vital role in decision making selecting a country for study abroad. Moreover, international students from advanced countries and less economically developed countries have different intentions and reasons behind their mobility to take part in short-term or long-term educational programs. Due to their diverse relocation motives, the international students can be categorized as short-term “non-degree” and academic tourists, program students (long-term) “degree students” and free movers (Solaun, 2003). The factors that influence the categorization of international students are mainly associated with the motivation of international students toward foreign study programs like prioritizing their educational or social goals.

The sociocultural factors that absolutely predict the inclination to foreign education include respect for diverse cultures, excitement to meet new people and ideas, concentration in reading and writing, interaction with diverse people in the university, and participation in co-curricular activities (Sideli et al., 2015).

Yasmin et al. (2021) contended that the views of the old students of the concerned universities perform a vital role in the motivation level f the prospective students. Prior students’ satisfaction level with the service quality of the institutions corroborates the motivation of the new applicants. Another study by Kim and Cocks (2021) wobbled the importance of the local built environment of the host countries to attract and retain international students in China. They argued that the quality of life offered by the host country plays a metamorphosis role in the motivation of the international students. Moreover, understanding languages play a vital role in the attraction and motivation of prospering students. Li et al. (2020) found that multilingual and transnational networks construct a gateway in the shifting paradigm of students toward China. In the Chinese mainland, technology will reshape the history of universities. Students’ ambition to engender the new wave of technology is really widespread. The attainment of the elusive technology understanding motivates the influx of international students toward the market in China (Murphy, 2020).

Generally, the research on pre-enrollment expectations, goals, and motives of students put great stress on personal differences in the attitude of students toward their diverse beliefs about foreign education rather than considering the influence of students’ economic, social, and cultural backgrounds. It reveals that the attitudes and belief systems of individuals can strongly affect the actual experience of students. This direction may point out the significance of applying the theory of motivation to study students’ decisions of acquiring foreign education.

## Materials and Methods

### Data Collection

**FIGURE 1:**
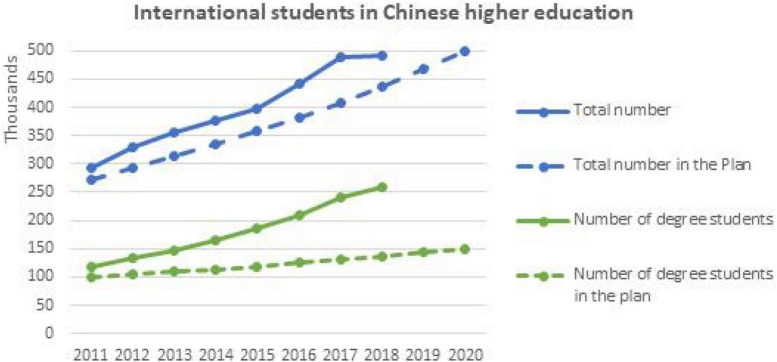
International students statistics.

**FIGURE 2:**
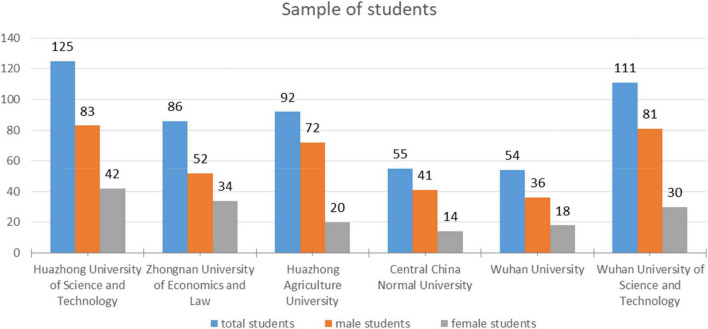
Sample attributes.

We collected the data for this study from the master’s and Ph.D. students enrolled in seven different universities in Wuhan, China. The justification for this selection is the consideration of this city as a Chinese educational hub due to the existence of numerous significant state-level colleges. Our sample includes 321 Asian students, 176 African students, and 150 European and American students (see Figure 1). This resulted in a sample size of 647 international students. Twenty-nine questions, however, were either incomplete or wrong. As a result, our final sample contains 618 valid questionnaires for data analysis. All of the selected universities are part of Project 211, which intends to build 100 significant Chinese universities for the twenty-first century, and thus receive preferential treatment and financial support from the Chinese Ministry of Education. The sample included all male and female foreign students enrolled in Master’s and Ph.D. programs. The researcher went to the universities in the sample to self-administer the questionnaire. The questionnaire was carefully filled out by the respondents. Foreign students’ participation in the poll was fully optional and anonymous. Details of the selected universities and the description of the sample size is given in Figure 2.

This study was carried out using the survey research approach. This approach has the best level of generalizability when it comes to representing a bigger population. The collected data has a better understanding of the relative characteristics of the study’s population. Because of the larger representative sample size obtained through this approach, it is typically easier to publish statistically vivid conclusions than when using alternative data. Procedures for gathering the examination of many variables can be carried out effectively by said research. Furthermore, for scientific research investigations, the survey approach is ideal since it provides a verified stimulus to all research participants (Douglas and Douglas, 2006).

**FIGURE 3:**
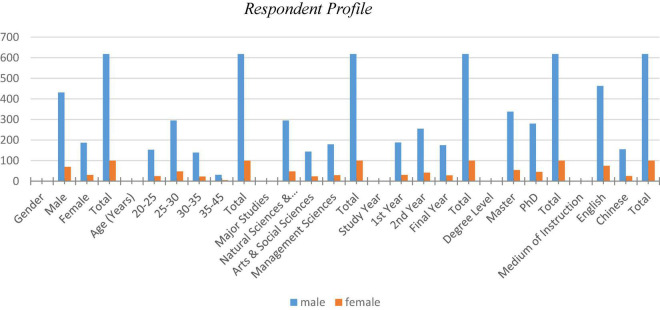
Respondent profile.

Figure 3 includes information on various demographic characteristics of the sample respondents to aid in a better comprehension of the data in this study.

The foreign students’ medium of instruction is listed below.

### Instrument of Research

We organized a questionnaire to examine the motivational factors of the sampled students among the Chinese universities. The research instrument of this study was mainly divided into four parts. The first part asked questions from respondents about such personal attributes as gender, nationality, degree level, the name of the university, and medium of instruction. The second part of the questionnaire was designed to explore the deciding motivational factors of global students to study in China. The third part of the questionnaire was developed to explore the service quality dimension of sample Chinese universities. Subsequently, the last part of the questionnaire explores the overall satisfaction of global respondents from their practice of studying in China. The details of each dimension construction are given below.

(I) Academic and Education Quality, this dimension consisted of nine items that are mainly relevant to the motivational factors of foreign students’ mobility such as acquiring a foreign degree, receiving quality education (Min et al., 2012b), completing the degree within due time, to get better teaching and research facilities (Zeeshan et al., 2013), to get the job due to upgrade their education (Min et al., 2012b), and increase in image and prestige at home country by holding a foreign degree.

(II) Personal and Financial Security, this dimension contained five items related to came China due to poor law and order situation in the home country, chose China because of safety reasons (Zeeshan et al., 2013), poor economic and employment conditions in the host country were the reasons to came to China, because of the reasonable amount of scholarship and came to study in China due to unemployment condition in the domestic country.

(III) Career and Migration contained five items to explore the motivational factors such as the opportunity to get a promotion in the current job due to study in China, finding a part-time job/business along with studies (Zeeshan et al., 2013), can gain the chance of English teaching experience while studying here, intend to find a job in China after the completion of degree and want to settle in China permanently (Min et al., 2012b).

(IV) Pleasure and Experience consisted of seven items intended to discover the motivational factors such as selecting China to study abroad due to having many tourist attractions, meeting people from different nationalities, removing the boredom from routine life, experiencing a different culture, to get away from stressful situations in the home country, to enjoy life as a foreign student and to come here in pursuit of an ideal life (Min et al., 2012b).

Other Motivational Factors were added to the questionnaire that includes six general items such as coming to China because funding agency/institution has sent here, moving to China because others (family and friends) invited to come here and helped in getting admission (Zeeshan et al., 2013) and came here to learn the Chinese language.

### Validity and Reliability

To extract eigenvalues and the variance explained by each component the principal components analysis technique was used.

**FIGURE 4:**
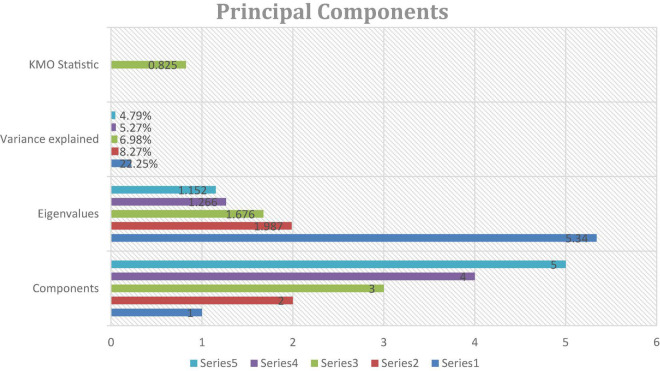
Principal components.

Figure 4 explains the Eigenvalues and the variance explained by each of the components extracted through the principal component analysis. Eigenvalue explains how many dimensions or how many components could be extracted from a set of data. The benchmark for the Eigenvalue is usually a value greater than or equal to 1. The eigenvalues reported above signify that all of our five dimensions qualify the criteria as eigenvalue in all cases is greater than 1. The first component has the highest level of eigenvalue and explains the largest amount of variance of 22.25% which could be interpreted as the 22.25% of the variance in the student’s motives to study in China is explained by our first component. Subsequently, the second principal component has the eigenvalue of 1.98 and explains 8.27% of the variance in the motivation of the foreign students. The third and fourth principal component explains 6.98 and 5.27% of the motivation respectively. While, our last principal component explains the smallest portion of the variance in foreign students’ motives to pursue their higher studies in China. Moreover, the value of the KMO statistic of sampling adequacy is 0.825. The usual cut-off point for this statistic is at least 0.50 so this outcome suggests that our sample is adequate for principal component analysis.

### Principal Component Analysis and Reliability Analysis of Motivational Aspects to Study in China

The forthcoming analysis outlines the factor or principal component analysis of the motivational factors for prospective international students in China. Further, it provides the dimension-wise and overall reliability score of the motivation part of the questionnaire. The results confirm the validity and reliability of the constructs in gauging various aspects of foreign students’ motives to pursue a study program in Chinese HEIs.

Table 1 presents the factor analysis of foreign students’ motivation along with the reliability analysis of each dimension. It is important to mention that if any factor or item has a factor loading of less than 0.3 it shows that this particular item is not contributing meaningfully toward the component to which it belongs. The overall Cronbach’s alpha of all the motivational factors is 0.843 which is quite acceptable and indicates that 84% of the variance explained by all the motivational dimensions is true variance. Thus, we can claim that the items are reliable and internally consistent. Bartlett’s Test of Sphericity statistic is also substantial at a *p*-value of 1%.

**TABLE 1:** Principal component analysis of foreign students’ motivation to study in China.

Motives/Items	Reliability statistic (alpha)	Factor loading
**1.Quality education**	0.635	
The certificate or degree conferred by my Chinese university would help me to get a job easily.		0.421
I planned to pursue in China and I want to earn a foreign degree.		0.369
I wanted to study in China so that I could finish my degree on time.		0.361
I chose to study at a Chinese university because its degree will increase my image and prestige at home country and abroad		0.349
**2.Personal and financial security**	0.674	
I came to China because of poor law and order situation in my country.		0.613
I opt to come to China because of poor economic and employment conditions in my country.		0.573
I came to study in China because I was unemployed.		0.476
I decided to move to China because of the reasonable amount of scholarship		0.419
I chose China as the country of my study abroad because it is a safe place.		0.410
**3.Career and migration**	0.628	
I intend to settle in China and do not want to go back.		0.540
I intended to pursue study in a country where I can gain English teaching experience while studying.		0.527
I prefer china to study because I thought that I will be able to find a part time job/business along with my studies.		0.497
After finishing my degree, I aim to work in China.		0.472
I chose to study in China because after the degree completion I will be able to get promotion in my current job.		0.375
**4.Pleasure and experience**	0.731	
I came to China to get away from the harsh surroundings in my own country.		0.597
I came here because I get bored from the routine life and was looking for some change.		0.545
I came to China to enjoy my life as a foreign student.		0.513
I chose China as the country of my study abroad because it is a vast country and has many tourist attractions.		0.504
I came to study in China in pursuit of an ideal life.		0.453
I came to here because I believed that here I can meet people from different nationalities.		0.399
**5.Other motives**	0.646	
Others (family and friends) compelled me to relocate to China.		0.570
I came to China because someone from my family or friends was studying here and he/she helped me in getting admission.		0.634
I chose to study in China because the Chinese value system matches with my personal beliefs.		0.417
I am studying in China because my funding agency/institution has sent me here.		0.512

*Bartlett’s Test of Sphericity: 3519, df = 276, p < 0.001.*

### Empirical Strategy

We performed item-wise, dimension-wise, and variable-level analyses to gain a thorough understanding. A number of dimensions to a few sample variables give adequate information as much as was available in the broader data collection (Mazziotta and Pareto, 2018). Given that the results of our survey data cover a variety of dimensions of service quality; the application of PCA allows us to transform a larger collection of variables into a smaller set with minimal information loss. In addition, an ANOVA is used to determine significant difference.

## Data Analysis and Interpretation

It is important to determine the most important dimensions which describe the key inspiring factors for foreign students to study in China. This section discusses the descriptive results of the motivational dimensions, further it provides a ranking of the motivational constructs based on the mean score of respondents. Later, each motivational dimension is ranked based on the regional category of international students. This section ends with the ANOVA to check whether or not a statistically significant difference exists between the motives of foreign students.

Table 2 reveals the descriptive statistics of the motivation dimensions as well as the aggregate motivation score of the foreign students.

**TABLE 2:** Descriptive statistics of motivation dimensions.

Dimension	Mean	SD	Frequency	Percent (%)
Academic and education quality	3.75	0.58	463	75
Personal and financial security	2.72	0.77	336	54
Career and migration	2.76	0.74	341	55
Pleasure and experience	3.25	0.72	401	65
Other motivational factors	2.99	0.65	368	60

**FIGURE 5:**
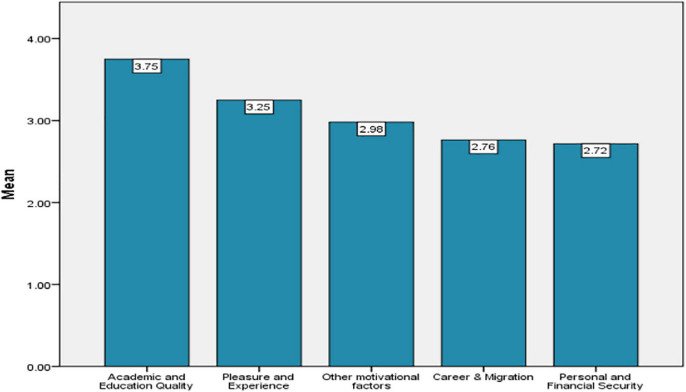
Mean score of the motivation dimensions in ascending order.

Figure 5 demonstrates the mean score of the motivation dimensions in ascending order. The graph clearly reveals that, to Obtain Quality Education and Pleasure and Experience were the key Motivational Aspects. Whereas, Personal and Financial Security got the least favorable response from the foreign students and were ranked lower among all the motivational dimensions.

The Table 3 presents the region-wise mean scores and ranking of foreign students on each motivational dimension. Asian students show a higher ranking on academic quality, financial security, and career and migration, while European students exhibit a higher ranking on the pleasure and experience dimension. Table 4 presents the region-wise analysis of variance in the motivational factors for foreign students to go after their higher education. The *F*-statistics and the corresponding significance level reveal that there are significant differences among foreign students coming from various regions of the world on all the motivation dimensions except Other Motivational Factors. Similarly, there is a shred of evidence of the statistical difference in the Overall Motivation level of students coming from Asia, Africa, and Europe. This outcome entails that the region has a significant effect on the preferences and motives of foreign students to pursue their higher education abroad.

**TABLE 3:** Region-wide differences in the motivation of foreign students.

Dimensions		N	Mean	Rank	SD
Academic and education quality	Asian	325	3.85	1	0.56395
	Africans	232	3.71	2	0.50864
	Europeans	61	3.35	3	0.71807
Personal and financial security	Asian	325	2.86	1	0.80265
	Africans	232	2.60	2	0.70100
	Europeans	61	2.37	3	0.68817
Career and migration	Asian	325	2.84	1	0.76939
	Africans	232	2.66	3	0.66169
	Europeans	61	2.76	2	0.83890
Pleasure and experience	Asian	325	3.35	2	0.73837
	Africans	232	3.07	3	0.66034
	Europeans	61	3.39	1	0.66615
Other motivational factors	Asian	325	3.02	1	0.68949
	Africans	232	2.96	2	0.56163
	Europeans	61	2.83	3	0.70685

**TABLE 4:** Results of analysis of variance (ANOVA) using foreign students region as the category variable.

Dimensions		Sum of squares	Degree of freedom	Mean Square	Frequency	Significance
Academic and educational quality	In between groups	13.157	2	6.579	20.882	0.000
	Within groups	193.744	615	0.315		
	Total	206.901	617			
Personal and financial security	Between groups	17.329	2	8.664	15.196	0.000
	Within Groups	350.662	615	0.570		
	Total	367.991	617			
Career and migration	Between groups	4.262	2	2.131	3.911	0.021
	Within groups	335.162	615	0.545		
	Total	339.424	617			
Pleasure and experience	Between groups	11.581	2	5.790	11.714	0.000
	Within groups	303.996	615	0.494		
	Total	315.577	617			
Other motivational factors	Between groups	2.073	2	1.036	2.482	0.084
	Within groups	256.871	615	0.418		
	Total	258.944	617			
Overall motivation	Between groups	7.135	2	3.567	12.271	0.000
	Within groups	178.790	615	0.291		
	Total	185.925	617			

There are contradictory arguments about the nature of pre-enrollment expectations and their subsequent role in affecting the satisfaction from the actual study experience. These factors are important to identify the students who have a strong will to acquire foreign education. In addition, the studies indicate the two different directions of the positive pre-enrollment expectations of students. The first direction suggests that undue positive expectations have unfavorable effects on the adaptation of students abroad. The second direction suggests that the pre-enrollment positive expectations have favorable effects and it reinforces the likelihood of the students to take part in foreign education programs. These directions associate with the diverse influence of positive expectations and therefore, permit further research in this area (Goldstein and Kim, 2006).

Foreign students are considerably different in terms of their motives to opt for an overseas destination for their studies abroad. Zeb et al. (2021) postulate that prior expectations of the students will define their resulting level of satisfaction. Chirkov et al. (2008) proposed that motivation to study abroad significantly helps foreign students in adaptation to the new environment. Likewise, Min et al. (2012b) suggested that higher education institutions must account for the motives of the students while devising their educational programs to increase the satisfaction levels of foreign students.

If the pre-enrollment expectations of students meet the level of expectation or intrude upon positively (for instance, the output is positive and even beyond the expectation) then it affects the overall satisfaction of students positively with their experience of the study abroad program. Though in a similar case where the expectations meet negatively or being violated negatively (for instance, the output is even more negative than expected) then it influences the student negatively in the terms of adjustment issues in academics, psychological problems, and sociocultural problems to adjust in a foreign country (Martin et al., 1995).

Furthermore, Berno and Ward (2003) examined a similar phenomenon in Asian students studying in New Zealand. Based on their findings, they postulate that in general Asian students came to the foreign country with the most optimistic pre-enrollment expectations rather than their experience of living abroad. The most negatively violated expectations relevant to lack of communication with the natives, discrimination, less emotional and social support, language barrier, and problems in dealing with the rules and regulations established by the New Zealand government. These negative outcomes made a difference between the pre-enrollment expectations and the real living experience abroad which resulted in depression, maladjustment, adaptation, and academic problems. Thus, the reality-based pre-enrollment expectations are associated with better positive adaptation to a foreign country. Therefore, studies defining negative and positive disconfirmed expectations specify that pre-enrollment expectations strongly affect the students’ adjustment abroad. A positive attitude (always be determined and ready to adjust) toward the new situation is helpful for foreign students. Moreover, conscious efforts of preparing oneself to face the challenges of foreign education resulted in reality-based expectations such as being mindful of language problems and other adjustment issues in the foreign country. Thus, international students need to keep realistic expectations and develop suitable strategies to cope with the challenges (Lauermann, 2015).

The foreign students’ adaptation to a new place is mainly linked to the likelihood of participating in the foreign study program. Goldstein and Kim (2006) showed in their study results that American undergraduate students who got a chance to participate in short-term study abroad program expressed the most positive expectations to study abroad such as excitement for expected enjoyment of their experience as well as an eagerness to meet new people, had less fear of the degree completion, much interested in learning of foreign language, and less biased toward racial equality rather than the students who got no opportunity of foreign education during their study period. The sociocultural beliefs and expectations made differences among the participants and non-participants of the study abroad program in logical way.

In addition to the pre-enrollment expectations, the experience of living abroad may also affect the set goals of students in participating in the foreign education program. Kitsantas (2004) conducted a study on college students in America. The findings revealed three types of effects as students became more willing to know about the host county’s culture, became determined to achieve academic goals like foreign language learning, and were more interested in the subject offered in the short-term study abroad program. Lastly, students became more connected with friends who were taking part in the foreign education program and establish a bond with the host country’s cultural inheritance. The above findings propose that the set goals or expectations of students before going to a foreign country greatly influence their experience of learning in the foreign education program.

Chirkov et al. (2007) conducted a study to examine the motivational factors of Chinese international students in Canada and Belgium by using self-determination theory (SDT). The framework of theory recommends that the similar behavior of international students is motivated by diverse reasons. These different reasons behind the motivation to take part in a study abroad program may range from self-determined and independent to forced and fully controlled by the outer environment (Ryan and Deci, 2000). The completely independent and self-determined motivations are basically linked with the most positive output of students like, mentally relaxed, good performance, and better learning. However, motivated behaviors by the outer environment are associated with more negative output. The students who experience the more independent type of motivation are the most satisfied because of the fulfillment of their basic requirements of autonomy, competence, and relatedness. Whereas autonomy is related to self-determination of an individual’s behavior, competency is related to effective interaction with the social environment, and relatedness is linked with the establishment of more positive rapport with others (Schmuck et al., 2000). In adding to what extent personal goals relate to basic needs, the particular goals are classified into intrinsic and extrinsic goals. Intrinsic goals are mainly relevant to the significant relationship, individual growth, and social support, while, extrinsic goals are relevant to financial security, positive reputation, and physical attraction. As with autonomous motivation, the intrinsic goals enhance psychological relaxation and efficiency in working (Kasser and Ryan, 1993; Ryan and Deci, 2000).

Thus, a motive-focused educational program will not only be able to attract more foreign students but will also be able to enhance their satisfaction with the services provided by these HEIs. Based on these arguments the present study aims to figure out the broad motivational factors of foreign students to opt for Chinese places as a study hub and to what degree these motives relate to their overall satisfaction.

## Conclusion

The study findings led to various conclusions as a result of an investigation of international students’ evaluations of the Chinese universities’ service quality. These findings not only illustrate the current situation of international higher education programs in Chinese universities, but they also merit consideration from the respective authorities. These universities will be transformed into long-term knowledge cultivation hubs of international standings.

A detailed insight into the extant literature clarifies that international students have a vast set of intrinsic as well as extrinsic factors which motivate them to register in a study abroad program. Though, mainly the push and pull factors based on the social, economic, and cultural factors largely shape the decision of students, yet, personal factors such as the openness to experience and risk appetite of individuals also play a significant role in their foreign study decisions. The highly discussed factors in the literature about motivational factors of foreign students to study abroad were to get better academic and career prospects, migration, experience a different culture, tourism, and seek enjoyment and fun. However, the region and age of foreign students were also found to influence the motivational aspects of these students. As students from advanced countries mainly prefer to enroll in a short-term degree or exchange program with the key motive to seek pleasure and enjoyment from their short-term stay in a country abroad. While students from developing countries were inclined to get enrolled in long-term degree programs and academic motives were quite dominant in their abroad study decisions. An exhaustive survey of foreign students’ motivation reveals that academics have mainly investigated the motivational factors of foreign students registered in developed countries. Yet, recent positive growth in the number of foreign students in China and the emergence of China as a key international market for higher education makes it crucial to know the dynamics of the decision of these foreign students. Moreover, past empirical studies on the subject also provide footings to understand the motivational factors of a massive number of foreign students coming to China to pursue higher education and thus aid in constructing a tool to observe the underlying phenomenon.

Some academics argued that the motivation level of foreign students can help in adapting to the new environment and HEIs shall account for these motives in order to devise a more effective academic program. The pre-expectancy theory and expectation disconfirmation framework also support this argument. Additionally, the studies on the motivation of foreign students also indicate that foreign students have a range of academic and non-academic motives for pursuing their studies abroad where some students are very ambitious to upgrade their existing knowledge and skills while others may prefer to pursue non-academic motives. Thus, by linking the ideas put forward in the extant literature on motivation we conjecture that the motivational factors of foreign students are important to be understood to perceive their resulting satisfaction.

### Policy Implications and Future Directions

Against the backdrop of the massive Chinese government’s substantial financial and policy support for the long-term globalization of China’s higher education system. As a result, we use a bottom-up statistical technique to handle this research and found that different academic and non-academic factors motivate foreign students toward Chinese universities. These findings postulate many policy implications for the Chinese HEIs. Firstly, the understanding of the motivational factors of the foreign students provides them a ground to make new policies according to the factors. This revision of policies not only grants them more recognition but also is a reason for their high ranking. Secondly, the findings give a way to differentiate the main motives of students’ satisfaction and highlight the importance of academic and non-academic factors among the students. This understanding will improve and strengthen the Chinese education system. Last but not the least, practitioners will be able to learn that do the students of the different regions are motivated by the same factors or their region diversity affect their motivation too.

Our study provides new avenues for future research by highlighting that another study can be conducted on the comparison of motivational factors in China among undergraduate and post-graduate students and what kind of challenges are faced by Chinese universities to accommodate foreign students. We are leaving these questions for future research.

## Data Availability Statement

The raw data supporting the conclusions of this article will be made available by the authors, without undue reservation.

## Author Contributions

FY: writing original draft. SL: supervision. GS: review and funding. All authors contributed to the article and approved the submitted version.

## Conflict of Interest

The authors declare that the research was conducted in the absence of any commercial or financial relationships that could be construed as a potential conflict of interest.

## Publisher’s Note

All claims expressed in this article are solely those of the authors and do not necessarily represent those of their affiliated organizations, or those of the publisher, the editors and the reviewers. Any product that may be evaluated in this article, or claim that may be made by its manufacturer, is not guaranteed or endorsed by the publisher.
